# Prognostic Significance of PTEN Loss in Prostate Cancer: A Meta-Analysis of Gleason Grade and Clinical Outcomes

**DOI:** 10.3390/cancers17172862

**Published:** 2025-08-30

**Authors:** Filip Kisiel, Dougal Ferguson, Claire Hart, Mick Brown, Pedro Oliveira, Ashwin Sachdeva, Peter Gardner

**Affiliations:** 1 Department of Chemical Engineering, School of Engineering, University of Manchester, Oxford Road, Manchester M13 9PL, UK; filip.kisiel@postgrad.manchester.ac.uk (F.K.);; 2 Photon Science Institute, University of Manchester, Oxford Road, Manchester M13 9PL, UK; 3 Division of Cancer Sciences, University of Manchester, Manchester M13 9PL, UK; 4 Department of Pathology, The Christie Hospital NHS Foundation Trust, Manchester M20 4BX, UK; 5 Department of Surgery, The Christie Hospital NHS Foundation Trust, Manchester M20 4BX, UK

**Keywords:** PTEN loss, prostate cancer, Gleason Grade, risk stratification

## Abstract

Aims: Prostate cancer (PCa) presents ongoing challenges in differentiating aggressive from indolent disease using traditional biomarkers such as prostate-specific antigen (PSA). The Phosphatase and Tensin Homolog (PTEN), a key tumour suppressor involved in cellular growth regulation, is emerging as a promising biomarker for risk stratification. This meta-analysis aims to evaluate the prognostic significance of PTEN loss in PCa, particularly its relationship with Gleason grade groups (GG), as defined by the ISUP system, and clinical outcomes. Methods: A systematic review and meta-analysis of 16 studies encompassing 11,375 patients was conducted in accordance with PRISMA guidance. Studies included evaluated PTEN loss, stratified by hemizygous and homozygous deletions, and its association with GG and clinical endpoints such as biochemical recurrence and lethal progression. Pooled odds ratios (ORs) and hazard ratios (HRs) were calculated using a random-effects model. Results: PTEN loss was significantly associated with tumour aggressiveness. Compared to GG1 tumours, the odds of PTEN loss were markedly increased in Gleason GG 2 and 3(OR: 2.78, 95% CI: 1.95–3.61) and GG ≥ 4 (OR: 6.35, 95% CI: 5.37–7.33). Homozygous PTEN deletions were more strongly associated with high-grade tumours than hemizygous deletions. Clinically, PTEN loss was predictive of adverse outcomes, including increased risk of biochemical recurrence (HR: 1.78, 95% CI: 1.31–2.25) and lethal progression (HR: 2.57, 95% CI: 1.12–3.95). Conclusion: PTEN loss correlates with higher GG and poorer clinical outcomes in PCa. Incorporating PTEN assessment into clinical decision making could improve risk stratification, guiding early intervention strategies and identifying patients suitable for active surveillance.

## Simple Summary

Prostate cancer is one of the most common cancers in men. While current diagnostic tools can stratify risk, additional markers may help improve decision making, particularly in distinguishing between aggressive and less harmful forms of the disease. One such promising marker is Phosphatase and Tensin Homolog (PTEN), a gene that regulates cell growth. When PTEN is lost or deleted, it may indicate a more aggressive tumour. In this study, we analysed data from over 11,000 prostate cancer patients to explore how PTEN loss relates to tumour grade and patient outcomes. We found that PTEN loss, especially when both copies of the gene are deleted, is strongly associated with higher-grade disease and an increased risk of recurrence and death. These findings suggest that testing for PTEN loss may improve risk assessment and help identify patients who could benefit from earlier or more intensive treatment.

## 1. Introduction

Prostate cancer (PCa) is the second most common cancer in men globally, with incidence increasing annually since 2014, highlighting a need for improved diagnostic precision and personalised therapy [1,2,3].

Despite the use of clinicopathological variables such as symptoms, pathology results (Gleason Grade Groups (GG), number of biopsy cores, maximum percentage of core involvement) and serum prostate-specific antigen (PSA) levels for risk stratification, accurately differentiating between indolent and aggressive forms of cancer remains a major challenge [4]. PSA is the most utilised biomarker in clinical practice and is effective for monitoring disease progression; rising PSA levels are linked with disease recurrence or progression [4]. Although PSA testing effectively monitors PCa progression, it has limited diagnostic accuracy, contributing to significant overtreatment and under-diagnosis.

Despite stringent eligibility criteria for active surveillance, approximately 30% of patients initially categorised as low-risk are found to have more aggressive disease within 1–2 years [5]. However, many tumours identified through PSA testing do not require intervention. This highlights the critical need for additional prognostic markers to refine initial risk stratification and guide treatment decisions, so that truly aggressive tumours are identified early while patients with indolent disease are spared unnecessary intervention.

The GG system remains central to PCa prognosis, yet limitations in characterising tumour morphology persist. Tissue-based biomarkers are increasingly used to refine risk stratification. Among these, the PTEN located at 10q23.31, has emerged as a key tumour suppressor gene implicated in prostate cancer [4] and has been shown to distinguish indolent from aggressive tumours, thereby predicting disease progression [6,7].

**Figure 1:**
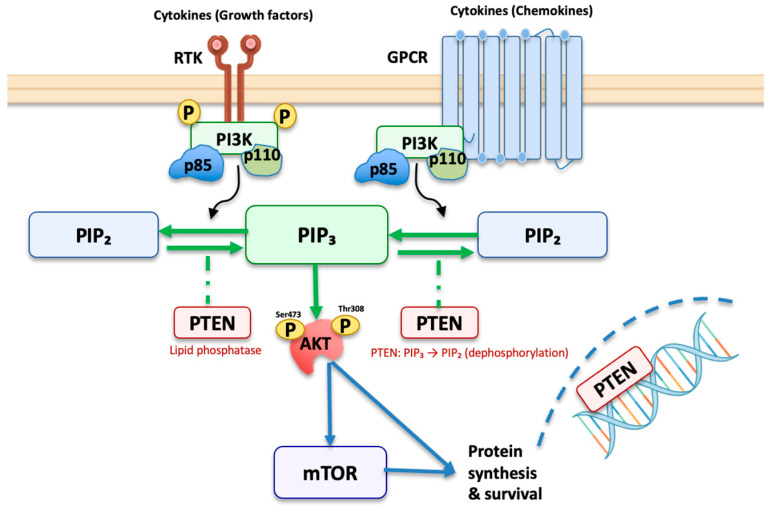
Growth factors and chemokines activate receptor tyrosine kinases (RTKs) and G-protein-coupled receptors (GPCRs), engaging class I PI3K (p85/p110) to convert PIP_2_ to PIP_3_ at the membrane. PIP_3_ recruits AKT, which is phosphorylated (Thr308 by PDK1, Ser473 by mTORC2) to drive mTORC1 dependent protein synthesis and survival. The lipid phosphatase PTEN reverses this step (PIP_3_ to PIP_2_) and hence restrains AKT-mTOR signalling. Reduced PTEN activity elevates PIP_3_ and amplifies the pathway. In the nucleus, PTEN also supports genomic stability and DNA repair. *Conventions:* green arrows, activation; dashed lines, inhibition. Abbreviations: RTK, receptor tyrosine kinase; GPCR, G-protein-coupled receptor; PI3K, phosphoinositide 3-kinase; p85/p110, PI3K regulatory/catalytic subunits; PIP_2_, phosphatidylinositol-4,5-bisphosphate; PIP_3_, phosphatidylinositol-3,4,5-trisphosphate; PTEN, phosphatase and tensin homologue; AKT, protein kinase B; mTOR, mechanistic target of rapamycin; Ser/Thr, serine/threonine; P, phosphate.

PTEN negatively regulates the PI3K/AKT/mTOR pathway by dephosphorylating PIP3 to PIP2, thereby maintaining cellular homeostasis [4,8]. Loss of PTEN function via deletion, mutation, or epigenetic silencing leads to unchecked PI3K signalling, AKT activation, and mTOR pathway engagement, promoting cell growth, survival, and proliferation [9,10]. PTEN also regulates non-PI3K pathways, influencing cell migration, angiogenesis, and genomic stability [11,12,13]. Beyond canonical signalling, PTEN deficiency promotes PCa metastasis through mechanisms such as EphA2 activation at serine 897, particularly facilitating PCa bone marrow invasion [12]. PTEN loss has also been linked to increased macrophage infiltration and TNF-α expression, further promoting tumour survival [14,15]. This is further visualised in Figure 1.

While other elements like tyrosine kinase receptors and upstream oncogenes may activate PI3K independently, PTEN loss remains a dominant driver of pathway dysregulation [9,16]. Importantly, crosstalk with androgen receptor and MAPK pathways adds complexity to PTEN-deficient tumour behaviour [8,17].

Clinically, PTEN loss correlates with aggressive PCa phenotypes and higher GG, yet its prognostic utility remains debated. Retrospective studies link PTEN loss in biopsies with adverse pathology and reduced survival outcomes [4,8,18,19,20,21,22]. However, not all studies have observed significant prognostic value for PTEN status once conventional predictors are accounted for, and thus no clear consensus has been reached on the magnitude or consistency of its effect. This uncertainty in the literature highlights the need for a comprehensive evaluation of PTEN’s prognostic significance. We therefore conducted a systematic review and meta-analysis to clarify the relationship between PTEN loss and prostate tumour aggressiveness (as reflected by GG) and to quantify its impact on clinical outcomes. We also aim to assess whether these data can guide real-world decisions (active surveillance triage, avoidance of focal therapy, and consideration of adjuvant/salvage treatment) in the discussion.

### 1.1. Methodology

This meta-analysis was conducted in accordance with the PRISMA (Preferred Reporting Items for Systematic Reviews and Meta-Analyses) guidelines (Prospero number CRD42024606420) [23]. A comprehensive literature search was performed in PubMed, Scopus, Web of Science, and Embase from database inception up to 1 November 2024, to identify studies evaluating the prognostic impact of PTEN loss in PCa. Studies were eligible if they reported on PTEN alterations (loss, heterozygous, or homozygous deletions) and examined these in relation to cancer severity indicators, specifically stratified by GG. For this study, histological aggressiveness is defined using the ISUP Gleason Grade Group system, which stratifies Gleason Scores as follows: Grade Group 1 (3 + 3), 2 (3 + 4), 3 (4 + 3), 4 (8), and 5 (9–10). The search strategy employed terms targeting disease context, PTEN gene alterations, tumour characteristics, and clinical outcomes. A detailed search strategy is attached to the Supplemental Table S1.

The primary outcome was the association between PTEN loss and prostate cancer severity, as assessed by GG. Secondary analyses investigated the significance of heterozygous versus homozygous PTEN deletions, verified by immunohistochemistry (IHC), fluorescence in situ hybridization (FISH), or sequencing.

Secondary outcomes assessed the prognostic role of PTEN loss in prostate cancer progression across the disease continuum from early recurrence to advanced stages. Clinical endpoints were categorised as recurrence-free survival (RFS) and lethal progression (LP). RFS was defined as a time from radical prostatectomy (or study entry) to the first occurrence of biochemical recurrence (PSA rise), distant metastasis, or death from any cause, whichever occurred first. Patients without an event were censored at the time of last follow-up. LP was defined as a time from radical prostatectomy (or study entry) to prostate cancer-specific death or the development of distant metastases. Deaths from causes unrelated to PCa were censored. To ensure a robust evaluation of PTEN as a predictive biomarker, these outcomes were stratified by PTEN status and assessed across different treatment contexts, including radical prostatectomy and androgen deprivation therapy. Additional endpoints of interest included biochemical recurrence-free survival and prostate cancer-specific mortality, offering further insight into the broader clinical implications of PTEN loss.

### 1.2. Data Extraction

Two reviewers independently extracted data from studies that met the inclusion criteria and were included in the meta-analysis. Extracted data included the following variables: Number of patients, Gleason grade, PTEN status (homozygous or heterozygous deletion vs. PTEN wildtype), method of PTEN assessment (e.g., IHC, FISH, sequencing), and primary outcomes such as lethal progression or recurrence free survival. Where reported, additional data on study-specific adjustments for confounders in the outcome analyses were also extracted.

### 1.3. Study Quality and Risk of Bias Assessment 

Study quality was assessed using the Newcastle-Ottawa Scale (NOS). Each study was independently evaluated by both reviewers across the NOS criteria, with studies scoring above 5 included in the meta-analysis. Domain-level risk of bias was additionally evaluated with the Quality in Prognosis Studies (QUIPS) tool (participation, attrition, prognostic-factor measurement, outcome measurement, confounding, analysis/reporting) and summarised with a traffic-light plot. Publication bias was evaluated using Egger’s regression test and visually inspected through a funnel plot for asymmetry. Discrepancies between reviewers in data extraction or quality assessment were resolved through discussion.

### 1.4. Statistical Analysis, Heterogeneity Assessment, Publication Bias

Heterogeneity was assessed using the I-squared (I^2^) statistic, with thresholds set at <25% (low), 25–50% (moderate), and >50% (high) [24]. A random-effects model was used for pooling the results when I2 > 50% to account for study heterogeneity, with the effect measure reported as a 95% confidence interval (CI); otherwise, a fixed-effect model was applied. Study weights were calculated by the inverse of the variance of each effect size. Sensitivity analyses were conducted by excluding each study in turn to assess individual influence.

Significance was tested using chi-square (df = k − 1) and Z-scores in subgroup analyses, with *p* < 0.05 considered significant. Analyses were conducted in Microsoft Excel (Microsoft Corp., Redmond, WA, USA) and Python (v3.12; Python Software Foundation)

Publication bias was evaluated using Begg and Mazumdar’s rank correlation and Egger’s regression tests, complemented by funnel plots. Begg and Mazumdar’s test provided an additional check for asymmetry.

## 2. Results

**Figure 2:**
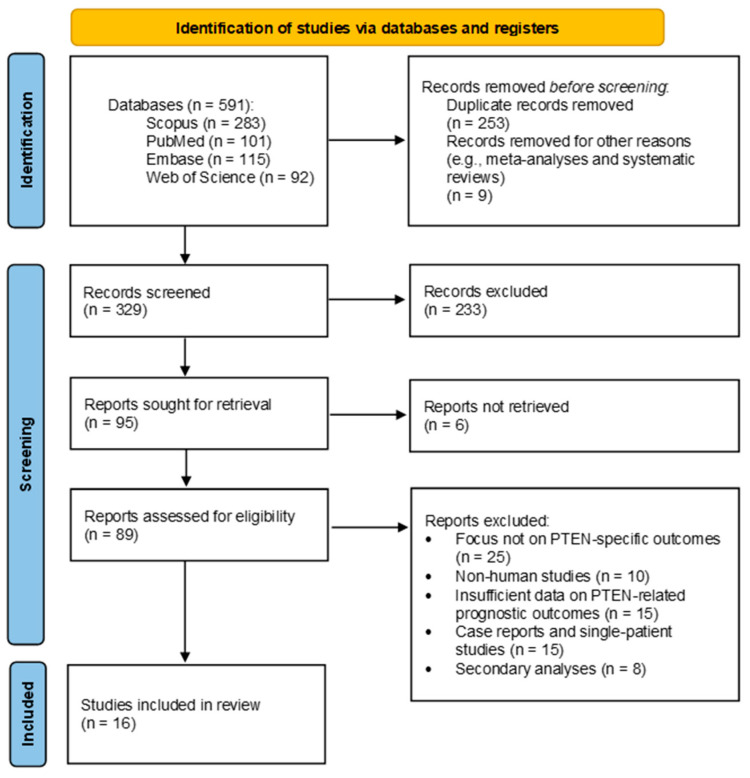
PRISMA Flow Diagram of Study Selection Process.

An initial search yielded 591 studies. After screening and selecting those that met the inclusion criteria, sixteen studies were included in this meta-analysis (Figure 2). All studies reported on the relationship between GG and complete PTEN loss versus wildtype (undeleted) PTEN status (Table 1). Additionally, four studies provided data differentiating between homozygous and heterozygous PTEN loss (Table 1), and all studies included specified outcome types (Table 2).

### 2.1. Quality Assessment

**Figure 3:**
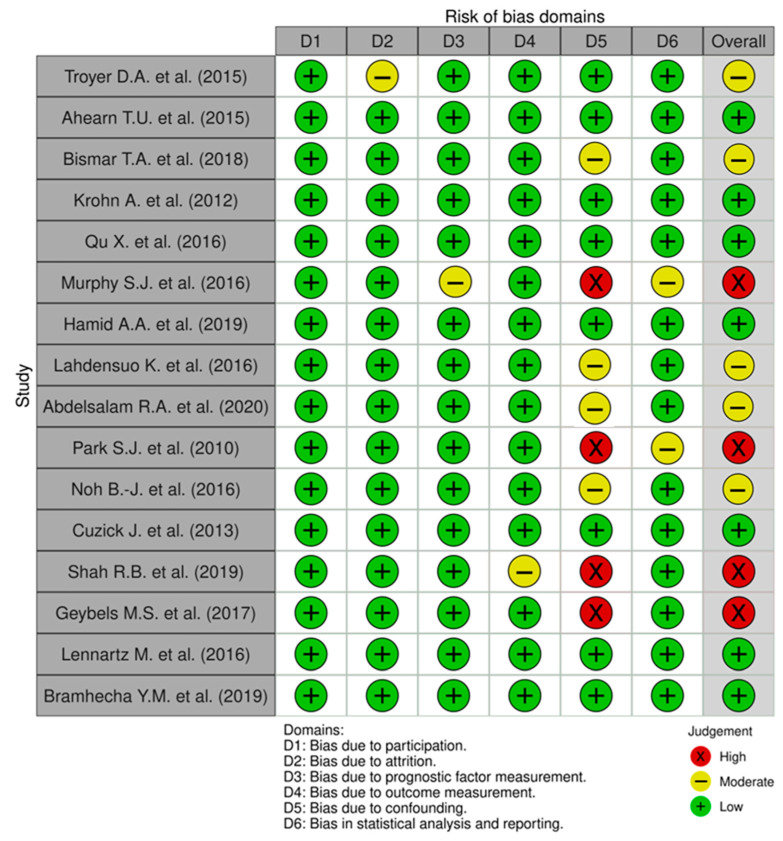
Traffic-light risk-of-bias assessment across included studies (QUIPS) [5,19,23,24,25,26,27,28,29,30,31,32,33,34,35,36]. Each row represents a study; columns D1–D6 are QUIPS domains: D1 participation, D2 attrition, D3 prognostic-factor measurement, D4 outcome measurement, D5 confounding, D6 statistical analysis & reporting. Colours indicate judgment (green low risk, yellow moderate, red high). The Overall column summarises domain-level judgments for each study [37].

Each study scored 6 or above on the NOS scale. The QUIPS traffic-light summary (Figure 3) indicated low risk for selection/participation and outcome ascertainment. There was occasional moderate risk for prognostic-factor measurement (assay/scoring variability) and attrition, and isolated moderate–high risk for confounding due to heterogeneous adjustment. Statistical analysis and reporting was largely low risk.

To evaluate the potential for publication bias, both Egger’s regression test and Begg and Mazumdar’s rank correlation test were conducted for all outcome measures. The *p*-values for Egger’s test were greater than 0.05 in all analyses, and similarly, the Begg and Mazumdar’s test did not indicate significant bias (*p* > 0.05). Additionally, funnel plots appeared largely symmetrical on visual inspection, with only slight asymmetry in some cases (see Supplementary Figure S1 for funnel plots). Overall, these findings do not suggest substantial publication bias.

### 2.2. Characteristics of the Included Studies

The meta-analysis included a total of 16 studies, encompassing data from 11,375 patients. These studies varied in their methodologies for assessing PTEN status, employing techniques such as IHC, FISH and whole-genome sequencing to determine PTEN loss or deletion.

### 2.3. Relationship Between PTEN Loss and Gleason Grade

**Figure 4:**
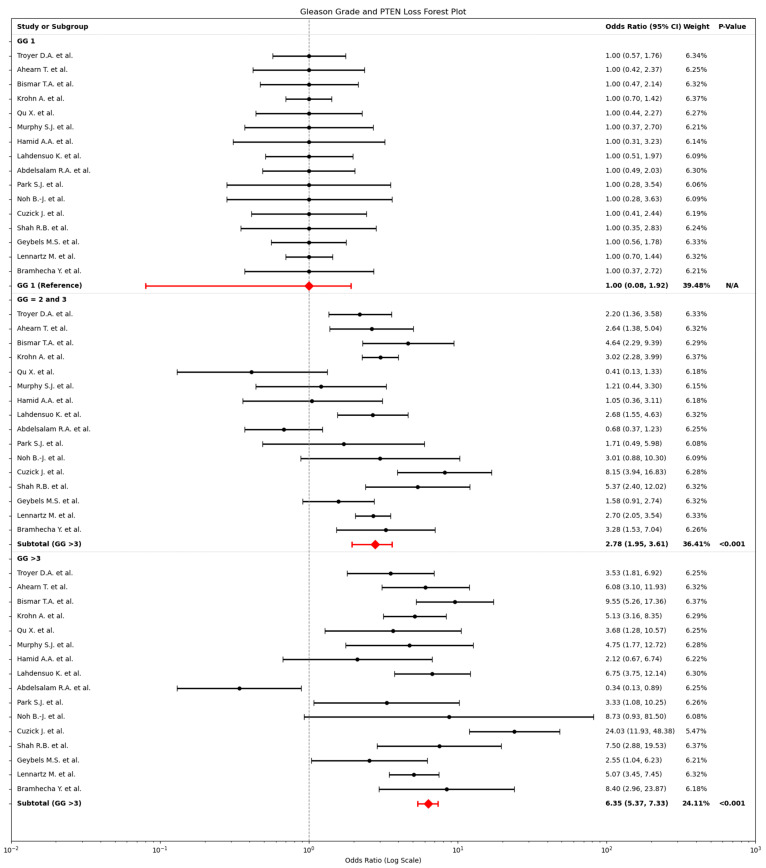
Forest Plot of PTEN Loss in Relation to Gleason Grades GG 1, GG2 and GG 3, and ≥4. Individual study odds (OR, black dots) and 95% confidence intervals (horizontal lines) for PTEN loss are shown for tumours with GG ≤ 1 (**top panel**), GG = 2 and 3 (**middle panel**), and GG ≥ 4 (**bottom panel**) [5,19,23,24,25,26,27,28,29,30,31,32,33,34,35,36]. GG1 is the reference category (OR fixed at 1.0 for orientation only; not meta-analysed). Red diamonds indicate the pooled OR for each subgroup. Gleason 2 and 3: OR 2.78 (95% CI 1.95–3.61), GG ≥ 4: OR 6.35 (95% CI 5.37–7.33).

The relationship between PTEN loss and Gleason grade (GG) in PCa was assessed using a random-effects model due to high heterogeneity (I^2^ > 90%) across studies (Figure 4). This analysis examined three GG categories: GG 1, GG 2 and 3 and high-grade (≥4). Odds ratios (OR) for PTEN loss were calculated for each category relative to the reference group GG 1, providing insight into how PTEN loss correlates with tumour grade. The results showed a significant increase in the odds of PTEN loss with higher GG, suggesting a link between PTEN status and tumour aggressiveness. Specifically, for GG 2 and 3, the odds of PTEN loss were 2.78 (95% CI: 1.95–3.61). For GG ≥ 4, the odds were 6.35 (95% CI: 5.37–7.33).

### 2.4. Relationship Between PTEN Loss and Intermediate Gleason Grades (2 and 3)

**Figure 5:**
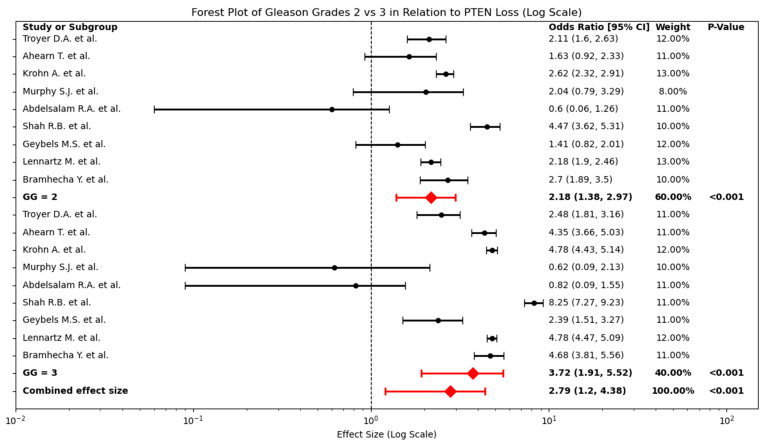
Forest Plot Comparing PTEN Loss in GG 2 vs. GG 3 in PCa. Individual study odds ratios (OR, black dots) and 95% confidence intervals (horizontal lines) for PTEN loss are shown separately for GG 2 (**top panel**) and GG 3 (**bottom panel**) [23,24,26,28,29,33,34,35,36]. Red diamonds indicate the pooled OR for each subgroup. GG 2: OR 2.18 (95% CI 1.38–2.97), GG 3: OR 3.72 (95% CI 1.91–5.52). The bottom-most red diamond shows the overall pooled OR across both subgroups: 2.79 (95% CI 1.20–4.38).

In addition to analysing PTEN loss across broad Gleason grade categories, a targeted comparison was conducted between GG 2 and GG 3 subgroups. The forest plot (Figure 5) demonstrates that PTEN loss is more strongly associated with GG 3 (OR: 3.72, 95% CI: 1.91–5.52) than with GG 2 (OR: 2.18, 95% CI: 1.38–2.97). The Z-score for this difference, calculated at −0.65, indicates that while there is a trend toward increased PTEN loss in GG 3, the difference is not statistically significant.

### 2.5. Relationship Between Hemizygous and Homozygous PTEN Loss in Relation to Gleason Grades

**Figure 6:**
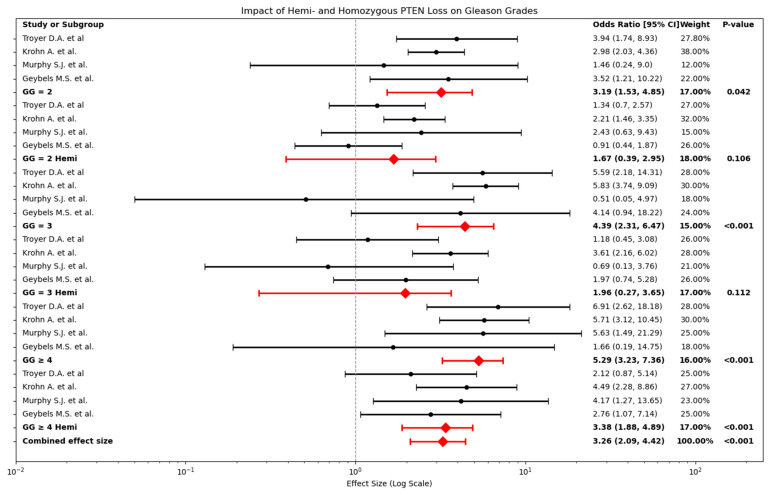
Forest Plot of Hemi- and Homozygous PTEN Loss in Relation to GG 2, 3, and ≥4 in PCa. Individual study odds ratios (black dots) and 95% confidence intervals (horizontal lines) are shown separately for homozygous (top row in each panel) and hemizygous (bottom row in each panel) PTEN loss across three GG: 2 (**left panel**), 3 (**middle panel**), and ≥4 (**right panel**) [23,26,28,34]. Red diamonds denote the pooled effect for each loss type and grade. GG 2 Homozygous PTEN loss OR: 3.19 (95% CI 1.53–4.85), Hemizygous PTEN loss OR: 1.67 (95% CI 0.39–2.95), GG 3 Homozygous PTEN loss OR: 4.39 (95% CI 2.31–6.47), Hemizygous PTEN loss OR: 1.96 (95% CI 0.27–3.65), GG ≥ 4 Homozygous PTEN loss OR: 5.29 (95% CI 3.23–7.36), Hemizygous PTEN loss OR 3.38 (95% CI 1.88–4.89). The bottom-most red diamond shows the overall pooled OR for any PTEN loss across all grades: 3.26 (95% CI 2.09–4.42).

Figure 6 illustrates the impact of hemi- and homozygous PTEN loss across Gleason grades 2, 3 and ≥4.

In GG 2, homozygous PTEN loss shows a significant association with increased tumour aggressiveness (OR: 3.19, 95% CI: 1.53–4.85, *p* = 0.042). When comparing homozygous PTEN loss to hemizygous PTEN loss in this subgroup, the Z-score is 1.42, indicating that the difference is not statistically significant, suggesting weaker confidence in the distinction. Hemi-deletions within the same subgroup show a weaker association with tumour aggressiveness (OR: 1.67, 95% CI: 0.39–2.95, *p* = 0.106).

For GG 3, homozygous PTEN loss exhibits a stronger association with tumour aggressiveness (OR: 4.39, 95% CI: 2.31–6.47, *p* < 0.001). The Z-score comparing homozygous and hemizygous PTEN loss in this subgroup is 1.78, again indicating that while there is a trend toward stronger associations for homozygous loss, the difference is not statistically significant. Hemi-deletions in this subgroup show a lower effect size (OR: 1.96, 95% CI: 0.27–3.65, *p* = 0.112).

In high-grade tumours (GG ≥ 4), homozygous PTEN loss shows the strongest association with tumour aggressiveness (OR: 5.29, 95% CI: 3.23–7.36, *p* < 0.001). When comparing homozygous to hemizygous PTEN loss in this subgroup, the Z-score is 1.47, indicating that the difference between these two types of PTEN loss is not statistically significant. Hemi-deletions in high-grade tumours also show a strong association (OR: 3.38, 95% CI: 1.88–4.89, *p* < 0.001).

The combined effect across all GGs (OR: 3.26, 95% CI: 2.09–4.42, *p* < 0.001) demonstrates a strong overall association between PTEN loss and tumour aggressiveness, with homozygous PTEN loss exerting a greater impact than hemi-deletions.

### 2.6. Clinical Outcomes and PTEN Loss: Results

**Figure 7:**
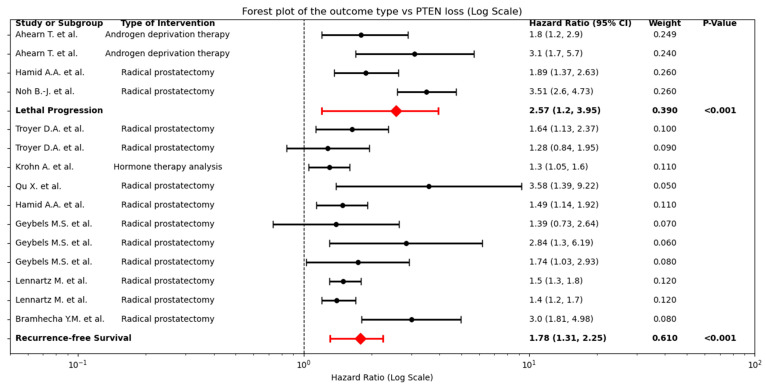
Forest Plot of Clinical Outcomes Stratified by PTEN Loss. Individual study hazard ratios (HR, black dots) and 95% confidence intervals (horizontal lines) are shown separately for lethal progression (**top panel**, pooled HR: 2.57 (95% CI 1.12–3.95) shown as a red diamond) and recurrence-free survival (**bottom panel**, pooled HR: 1.78 (95% CI 1.31–2.25) shown as a red diamond) [23,24,26,27,29,34,35,36].

Figure 7 shows that PTEN loss was associated with both recurrence and lethal progression. Specifically, the pooled analysis hazard of a recurrence-free survival event was increased in patients with PTEN loss (HR 1.78, 95% CI 1.31–2.25, *p* < 0.001). Likewise, PTEN loss conferred a higher hazard of lethal progression (HR 2.57, 95% CI 1.12–3.95; *p* < 0.001).

## 3. Discussion

This meta-analysis evaluated three aspects of PTEN loss in PCa: its association with higher Gleason Grade, the differential impact of homozygous versus hemizygous PTEN deletions on GG, and the relationship between PTEN loss and clinical outcomes such as lethal progression, biochemical recurrence, and cancer-specific survival. These analyses address key clinical challenges, particularly the debate over appropriate screening, detection, and treatment strategies for PCa as these can lead to either overtreatment of indolent disease or undertreatment of aggressive tumours [23,29,33].

### 3.1. PTEN Loss and Gleason Grade

Our meta-analysis reveals a strong correlation between PTEN loss and higher GG, reinforcing its role as a key marker of tumour aggressiveness and progression [28,35]. We observed that PTEN loss becomes increasingly prevalent in higher GG which reflects its association with more aggressive PCa phenotypes.

PTEN loss, primarily through PI3K/AKT/mTOR pathway dysregulation, facilitates tumour progression by promoting cell survival, proliferation, and resistance to apoptosis. This mechanism underlies its strong correlation with higher GG and aggressive clinical phenotypes [28,33].

By combining these molecular insights with clinical and histopathological findings, our meta-analysis highlights the central role of PTEN as both a tumour suppressor and a prognostic indicator in PCa. These results provide further evidence supporting the inclusion of PTEN status in the evaluation of PCa aggressiveness and its potential as a target for precision therapies.

### 3.2. Homozygous vs. Hemizygous PTEN Loss

In this subgroup analysis, we found that homozygous PTEN deletions are more frequently associated with higher-grade prostate tumours, particularly those with a GG of ≥4. Hemizygous PTEN deletion, showed a weaker and less consistent association with tumour grade. In contrast, homozygous deletions, lead to a complete inactivation of PTEN’s tumour-suppressor function. This loss facilitates sustained activation of the PI3K/AKT/mTOR signalling pathway, promoting angiogenesis and resistance to apoptosis [33,35]. The stepwise increase in PTEN loss, from intact to hemizygous to homozygous deletions, underscores its progressive role as a marker of malignancy [23,33].

Our analysis shows homozygous PTEN deletions are consistently more strongly associated with high-grade tumours and severe clinical outcomes compared to hemizygous deletions, aligning with previous findings linking complete PTEN inactivation to metastatic and treatment-resistant prostate cancers [28,38,39].

### 3.3. Clinical Outcomes and PTEN Loss: Discussion

Our meta-analysis revealed that PTEN loss is significantly associated with poorer clinical outcomes in PCa patients.

In the literature, several studies have demonstrated that PTEN status is linked to disease progression and helps identify patients at varying risks of death from PCa. For instance, Lotan et al. [40] reported that PTEN loss was associated with adverse pathological features and reduced recurrence-free survival. Krohn et al. [26] found that genetic deletion of PTEN was linked to tumour progression and early PSA recurrence in PCa. In patients undergoing active surveillance, PTEN status has been linked to disease progression; Lindberg et al. [41] suggested that PTEN loss could predict progression to more aggressive disease, indicating that PTEN assessment could inform decisions about early intervention. Additionally, Reid et al. [38] demonstrated that molecular characterisation of the PTEN gene locus helps identify patients at varying risks of death from PCa. Ferraldeschi et al. [42] showed that PTEN loss contributes to resistance to androgen deprivation therapies and newer treatments for castration-resistant PCa, such as enzalutamide and abiraterone. This resistance is believed to occur because the PI3K/AKT/mTOR pathway can act as an alternative survival route, bypassing the androgen receptor pathway that these therapies target [42]. Therefore, assessing PTEN status could enhance prognostic accuracy and guide therapeutic strategies in PCa management, both after radical prostatectomy and during active surveillance.

While our meta-analysis did not examine the differential impact of homozygous versus hemizygous PTEN loss, existing literature suggests that the extent of PTEN deletion influences clinical outcomes. One study [34] showed that patients with homozygous PTEN-deleted tumours had a significantly increased risk of recurrence compared to those with intact PTEN, whereas hemizygous loss was not significantly associated with recurrence.

As shown in this meta-analysis, PTEN can be an important marker in active surveillance (AS) considerations. Consequently, assessing PTEN status at the point of diagnosis may help identify ostensibly ‘low-risk’ tumours that are prone to progression and thus poor candidates for AS [4]. For example, Jamaspishvili et al. (2018) emphasised that PTEN loss can distinguish potentially aggressive GG 1–2 cancers that might otherwise be considered for surveillance [4]. In the Johns Hopkins AS cohort, PTEN loss was rare in GG 1 cancers that remained indolent but more frequent (though a minority) among cases reclassified early [43]. Cyll et al. in a more recent study showed that integrating PTEN status with other clinical variables improved risk stratification in men on AS [7]. Patients whose tumours had combined PTEN loss and DNA ploidy abnormalities were twice as likely to require definitive treatment. Incorporating these markers modestly improved predictive accuracy (increasing the CAPRA score’s c-index by 0.025) [7]. Practically, it may be suggested that a patient with otherwise low-risk features but PTEN deletion may benefit from closer monitoring or definitive therapy rather than AS.

### 3.4. PTEN as a Biomarker to Guide Focal Therapy and Adjuvant Radiation

Given its prognostic significance, PTEN loss is also being explored as a biomarker to tailor local therapy intensity. Patients with intermediate-risk disease or early adverse pathology (e.g., pT3a extracapsular extension) represent a heterogeneous group where management ranges from focal therapy or observation to multimodal treatment. Evidence suggests that PTEN status could inform these choices. For instance, men with GG 2 PTEN loss are more likely to have non-organ confined disease at prostatectomy (52% vs. 27% (*p* < 0.001) if PTEN intact) [22]. In one study, PTEN loss in a GG 2 biopsy independently doubled the odds of extracapsular extension or seminal vesicle invasion on final pathology (adjusted HR 2.46, *p* = 0.004) and improved the preoperative model’s predictive accuracy (AUC 0.67 vs. 0.61) [22]. This suggests that even intermediate cancers might be understaged if PTEN is deleted. Clinically, such data raises caution against less aggressive approaches (like focal ablation or limited brachytherapy) in PTEN-deficient tumours. A focal therapy strategy assumes the disease is confined and indolent. However, a PTEN-null lesion is more likely to represent an aggressive clonal focus with potential micrometastases or multifocal ex-tension. This argues for a surgery or whole-gland radiation instead. Although direct trials are lacking, PTEN could be used to refine patient selection for focal therapy or conservative management [22,44].

PTEN loss may also guide postoperative treatment decisions, such as the use of adjuvant radiation therapy (ART) in patients with pT3a or otherwise high-risk pathological features. Current clinical practice often involves either immediate ART or observation with early salvage radiation upon PSA rise for pT3 disease. Our meta-analysis confirmed that PTEN loss correlates with shorter time to biochemical recurrence and higher rates of progression to metastasis. A recent multi-institutional study of men undergoing salvage radiotherapy found that PTEN loss was an independent predictor of poor outcomes: any PTEN loss conferred significantly worse biochemical relapse-free and metastasis-free survival (HR 1.82; 1.12–2.96) despite salvage treatment [45]. Patients with homogeneous biallelic PTEN deletion had the highest risk of metastasis (HR 2.47; 1.54–3.95)) in that cohort [45]. In practical terms, if a post-prostatectomy patient has pT3a disease and known PTEN loss in the tumour, clinicians may favor adjuvant radiation (and/or systemic therapy) rather than AS to address the higher risk of occult residual disease. While no guidelines yet mandate PTEN driven therapy escalation, the evidence is increasing that PTEN status could be used to identify those ’intermediate’ or locally advanced cases who require earlier intervention. Future prospective trials are needed, but incorporating PTEN loss into adjuvant treatment decision algorithms is a logical next step given its association with recurrence risk.

### 3.5. Commercial Assays and PTEN Loss Assessment in Practice

Several commercial molecular assays are now available to assist in prostate cancer prognostication, and these tests incorporate PTEN’s impact on tumour behavior indirectly. The Decipher genomic classifier (a 22-gene expression panel), Myriad’s Prolaris test (a 46-gene cell-cycle progression score), Oncotype DX Genomic Prostate Score (GPS) (biopsy RT-PCR, 17-gene panel), and ProMark (biopsy protein-based 8-marker assay) are widely used examples. While none measure PTEN loss as a standalone readout, their composite scores reflect downstream consequences of PTEN inactivation among other factors. Tumours with PTEN loss often exhibit more aggressive gene expression profiles. PTEN deletions are associated with a ‘luminal proliferating’ subtype that tends to yield high Decipher scores (elevated metastatic potential), while the Prolaris panel captures aggressive proliferation signals that correlate with PTEN/PI3K pathway activation [46,47]. These assays are analytically validated and prognostic (e.g., Decipher predicts metastasis/adjuvant benefit; Prolaris, Oncotype DX GPS and ProMark stratifies AS vs. treatment) [48]. The latest National Comprehensive Cancer Network guidelines endorse the use of such tissue-based genomic tests during initial risk assessment, and list Prolaris, Oncotype DX GPS, Decipher, and ProMark as options that may be considered during initial risk stratification [49]. In contrast, PTEN IHC alone, as well as other single markers, is mentioned in the NCCN biomarker list but ‘not recommended’ for routine decision-making at this time [49]. This conservative stance likely reflects the fact that multi gene signatures provide a more comprehensive risk evaluation than any single biomarker. Nonetheless, clinicians are increasingly ordering these assays, and in doing so they are indirectly accounting for PTEN loss.

Our meta-analysis provides additional impetus, given the clear impact of PTEN loss on prognostic outcomes across many studies. At minimum, clinicians should be aware that currently available genomics tests are capturing elements of PTEN loss indirectly, and that a ‘high-risk’ molecular result likely signifies alteration in PTEN/PI3K pathway.

### 3.6. Limitations

This meta-analysis has several limitations. PTEN loss was assessed using diverse methodologies, including IHC, FISH and sequencing, which may have contributed to variability in the results with PTEN IHC showing sensitivities of 87% and 86% for hemizygous and homozygous deletions, compared to 65% and 97% for FISH [40,50]. Additionally, GG remain subjective, with inter-observer variability potentially affecting tumour classifications. While this analysis focused on PTEN loss, other molecular markers such as ERG and SPINK1, which frequently co-occur with PTEN loss, were not consistently explored, limiting a comprehensive evaluation of the molecular landscape. These factors likely contributed to the observed heterogeneity across studies.

Longitudinal studies tracking PTEN status over time in relation to GG changes would also be valuable in establishing survival and further explaining the role of PTEN in cancer evolution.

### 3.7. Future Research

Future research should prioritise integrating PTEN loss with other biomarkers, such as BRCA, CRISP3 expression, and additional signalling pathways, using multivariate Cox models to improve prognostic accuracy. Several studies have examined this is in a PCa prognostic [27,28,29,30,36]. The development of rapid, non-invasive diagnostic techniques, such as spectroscopy-based assays, could enhance the clinical applicability of PTEN analysis, particularly in real-time monitoring [51,52]. Additionally, longitudinal studies examining PTEN loss in relation to tumour progression would provide valuable insights. While current clinical guidelines primarily rely on GG, PSA levels, and tumour aggressiveness for decision-making, incorporating PTEN status into routine workflows could be beneficial in prognostic decision-making.

## 4. Conclusions

The significance of PTEN as a prognostic marker in PCa is well-established, with its loss consistently linked to aggressive tumour characteristics and poor clinical outcomes. This study underscores PTEN’s role as a biomarker, highlighting its strong association with higher GG and adverse prognoses. Assessing PTEN status has the potential to enhance prognostic accuracy, refine risk stratification, and guide therapeutic strategies, both after radical prostatectomy and during active surveillance. Incorporating PTEN evaluation into clinical practice could improve treatment decisions and ultimately enhance outcomes for patients with PCa.

## Figures and Tables

**Table 1:** PTEN Assessment Methods and Gleason Score Stratification Across Included Studies. GG 2 and 3 were not consistently distinguished across studies; where this occurred, the corresponding cells are merged. Greyed-out cells indicate cases where data on hemizygous or homozygous PTEN deletion were not reported. Values are number of patients (*n*).

Study ID	PTEN Assessment Method	GG	Undeleted, *n*	Hemi- Deletion, *n*	Homo- Deletion, *n*	Complete PTEN Loss, *n*
Troyer D.A. et al. (2015) [23]	FISH	<1	216	19	8	27
		2	178	21	26	47
		3	58	6	12	18
		≥4	43	8	11	19
Ahearn T. et al. (2015) [24]	IHC	<1	173			11
		2	338			35
		3	199			55
		≥4	168			65
Bismar T.A. et al. (2018) [25]	IHC	<1	120			15
		2	50			29
		3				
		≥4	103			123
Krohn A. et al. (2012) [26]	FISH	<1	647	32	36	68
		2	815	89	135	224
		3	185	33	60	93
		≥4	63	14	20	34
Qu X. et al. (2016) [27]	FISH	<1	63			14
		2	44			4
		3				
		≥4	11			9
Murphy S.J. et al. (2016) [28]	FISH and IHC	<1	35	6	4	10
		2	12	5	2	7
		3	17	2	1	3
		≥4	14	10	9	19
Hamid A.A. et al. (2019) [29]	FISH	<1	28			7
		2	38			10
		3				
		≥4	17			9
Lahdensuo K. et al. (2016) [30]	IHC	<1	243			18
		2	337			67
		3				
		≥4	100			50
Abdelsalam R.A. et al. (2020) [5]	IHC	<1	35			27
		2	69			32
		3	38			24
		≥4	27			7
Park S.J. et al. (2010) [31]	IHC	<1	8			12
		2	7			18
		3				
		≥4	10			50
Noh B.-J. et al. (2016) [19]	IHC	<1	8			11
		2	7			29
		3				
		≥4	1			12
Cuzick J. et al. (2013) [32]	FISH and IHC	<1	317			10
		2	144			37
		3				
		≥4	95			72
Shah R.B. et al. (2019) [33]	IHC	<1	63			8
		2	67			38
		3	21			22
		≥4	21			20
Geybels M.S. et al. (2017) [34]	IHC	<1	207	21	5	26
		2	141	13	12	25
		3	30	6	3	9
		≥4	25	7	1	8
Lennartz M. et al. (2016) [35]	IHC and FISH	<1	716			64
		2	1840			359
		3	454			194
	
		≥4	150			68
Bramhecha Y.M. et al. (2019) [36]	IHC and FISH	<1	52			9
		2	90			42
		3	37			30
		≥4	11			16

Abbreviations: FISH, fluorescence in situ hybridisation; IHC, immunohistochemistry.

**Table 2:** Summary of Included Studies Evaluating the Impact of PTEN Loss on Various Prostate Cancer Outcome Types.

Study ID	Outcome Type	HR (95% CI)	*p*-Value	Adjustments	Additional Findings
Troyer D.A. et al. (2015) [23]	RFS	1.64 (1.13–2.37)	0.009	Preoperative PSA, seminal vesicle invasion	PTEN homozygous deletion strongly linked to shorter RFS
	RFS	1.28 (0.84–1.95)	0.25	Preoperative PSA, seminal vesicle invasion	Hemizygous deletion not significantly associated with RFS
Ahearn T. et al. (2015) [24]	LP	1.8 (1.2–2.9)	Not specified	age, BMI, Gleason grade, and TNM stage	Complete PTEN loss associated with lethal progression
	LP (ERG-negative)	3.1 (1.7–5.7)	Not specified	Gleason grade and clinical stage	PTEN loss in ERG-negative cases shows strong association
Bismar T.A. et al. (2018) [25]	CSS	0.27 (0.18–0.42)	<0.0001	Gleason score, age	PTEN positivity significantly associated with improved CSS, strongest in non-ADT-treated cohort
	CSS	0.25 (0.16–0.39)	<0.0001	Gleason score, age	Reduced CSS risk for weak/moderate PTEN intensity
	CSS	0.43 (0.20–0.92)	0.029	Gleason score, age	High PTEN intensity associated with improved survival in multivariable model
Krohn A. et al. (2012) [26]	RFS	1.3 (1.05–1.60)	0.0158	Gleason grade, preoperative PSA level, pT stage	PTEN deletion independently predicts worse RFS
Qu X. et al. (2016) [27]	BCR	H3.58 (1.39–9.22)	0.008	Gleason grade, tumour stage, PSA	PTEN deletion significantly increases risk of BCR following radical prostatectomy
Murphy S.J. et al. (2016) [28]	BCR	HR not reported; recurrence rates reported instead.	Not specified	Not provided	PTEN deletion observed in ~60% of BCR cases, with ~80% recurrence in Gleason 7+ cases with PTEN loss
Hamid A.A. et al. (2019) [29]	MFS	1.49 (1.14–1.92)	<0.003	age, Gleason grade, and stage	Low PTEN expression strongly associated with metastasis in both continuous and dichotomous models
	OS	1.89 (1.37–2.63)	<0.001	Adjusted for age, Gleason grade, and stage	Lower PTEN expression linked to poorer overall survival outcomes
Lahdensuo K. et al. (2016) [30]	DSS	2.156 (1.169–3.976)	0.014	Univariate analysis	PTEN loss significantly associated with shorter DSS, especially in ERG-negative cancers
	Secondary therapy-free survival	2.782 (1.846–4.193)	<0.001	Adjusted for ERG/PTEN combined status	Higher likelihood of requiring secondary therapy post-radical prostatectomy with PTEN loss
Abdelsalam R.A. et al. (2020) [5]	BCR	OR 2.68 (0.98–7.33)	0.05	Adjusted for Gleason grade, path stage, surgical margin	PTEN-negative and ERG-positive cases show increased BCR risk
Park S.J. et al. (2010) [31]	PCa progression	HR not reported	0.019	None reported	Loss of PTEN expression significantly associated with elevated PSA levels, indicative of progression risk
Noh B.-J. et al. (2016) [19]	High-risk group (Low PTEN, High CRISP3)	9.979 (1.244–80.031)	0.03	Adjusted for subgroup risk (high-risk vs. low-risk). Low risk: Low PTEN and low CRISP3, high PTEN and low CRISP3, and high PTEN and high CRISP3 expression. High risk: Low PTEN and high CRISP3 expression	Low PTEN combined with high CRISP3 strongly associated with increased risk in cancer progression
Cuzick J. et al. (2013) [32]	PCa specific mortality	3.51 (2.60–4.73)	<0.0001	None specified in univariate; Adjusted in multivariate for Gleason score, PSA, and Ki-67 score	PTEN loss significantly predicts prostate cancer-specific mortality in low-risk patients
Shah R.B. et al. (2019) [33]	Intraductal carcinoma (IDC-P)	RR 4.993 (3.451–7.223)	<0.001	None	PTEN loss significantly associated with IDC-P, which shows the highest relative risk
	Cribriform Gleason Pattern 4	RR 2.459 (1.814–3.333)	<0.001	None	Strong association between PTEN loss and cribriform pattern, indicating poor prognosis
	Stromogenic PCa	RR 2.255 (1.634–3.112)	<0.001	None	PTEN loss linked with stromogenic PCa, a distinct morphological feature associated with worse outcomes
Geybels M.S. et al. (2017) [34]	RFS	1.39 (0.73–2.64)	<0.05	None specified	Hemizygous PTEN loss not significantly associated
	RFS	2.84 (1.30–6.19)	<0.06	None specified	Homozygous PTEN loss associated with increased recurrence risk
	RFS	1.74 (1.03–2.93)	<0.07	None specified	Any PTEN loss (hemi/homozygous) linked to a higher overall recurrence rate compared to PTEN intact cases
Lennartz M. et al. (2016) [35]	RFS	1.5 (1.3–1.8) for Intermediate vs. Low, 1.4 (1.2–1.7) for High vs. Intermediate	<0.0001	Multivariate adjustments including Gleason grade, pT stage, resection margin, and PSA levels	6q15 deletions and PTEN alterations significantly associated with poorer prognosis
Bramhecha Y.M. et al. (2019) [36]	BCR	3.00 (1.81–4.98) for PTEN deletion alone; 4.70 (2.12–10.42) for combined PTEN deletion and 16p13.3 gain	<0.0001	Adjusted for CAPRA-S score	PTEN deletion and 16p13.3 gain together strongly predict worse BCR, enhancing CAPRA-S-based stratification

Abbreviations: BCR, biochemical recurrence; BMI, body mass index; CRISP3, cysteine-rich secretory protein 3; CSS, cancer-specific survival; DSS, disease-specific survival; HR, hazard ratio; IDC-P, intraductal carcinoma of the prostate; MFS, metastasis-free survival; OR, odds ratio; OS, overall survival; pT, pathological tumour stage; PSA, prostate-specific antigen; RFS, recurrence-free survival; RR, relative risk.

## Supplementary Materials

The following supporting information can be downloaded at: https://www.mdpi.com/article/10.3390/cancers17172862/s1, Table S1: Search strategy; Figure S1: Funnel plots for the examination of small study effects for (a) GG 2 and 3 and (b) GG ≥ 4 for PCa.
