# Supplementary material for: Prognostic Significance of PTEN Loss in Prostate Cancer: A Meta-Analysis of Gleason Grade and Clinical Outcomes
Source: Cancers (Basel). 2025 Aug 30;17(17):2862. doi: 10.3390/cancers17172862 (PMC12427219; doi:10.3390/cancers17172862)
Supplement: Supplementary file 1 [file cancers-17-02862-s001.zip › cancers-3842281-supplementary.pdf]

Table S1. Search strategy.

Field	Search Terms	Boolean Operator Within Field	Boolean Operator Between Fields
**Disease**	"Prostate cancer"[Title/Abstract] OR "prostatic neoplasms"[MeSH Terms] OR "prostate carcinoma"[Title/Abstract]	OR	AND
**Gene (PTEN-related)**	"PTEN"[Title/Abstract] OR "phosphatase and tensin homolog"[Title/Abstract] OR "PTEN loss"[Title/Abstract] OR "PTEN deletion"[Title/Abstract] OR "PTEN mutation"[Title/Abstract]	OR	AND
**Tumour Characteristics**	"Gleason score"[Title/Abstract] OR "tumor grade"[Title/Abstract] OR "histopathological grading"[Title/Abstract]	OR	AND
**Outcomes**	"prognosis"[Title/Abstract] OR "survival"[Title/Abstract] OR "metastasis"[Title/Abstract] OR "recurrence"[Title/Abstract] OR "progression-free survival"[Title/Abstract] OR "overall survival"[Title/Abstract]	OR	AND

Figure S1. Funnel plots for the examination of small study effects for (a) GG 2 and 3 and (b) GG ≥ 4 for PCa. Figure A includes studies reporting outcomes for GG 2 and 3, while Figure B includes studies focusing on GG ≥ 4. Blue dots represent observed study estimates; orange circles indicate imputed studies added via the trim-and-fill method. The vertical lines indicate the combined effect size (green) and the adjusted effect size accounting for potential bias (red), both with 95% confidence intervals. All studies were also examined with the egger's test showing $p > 0.05$.

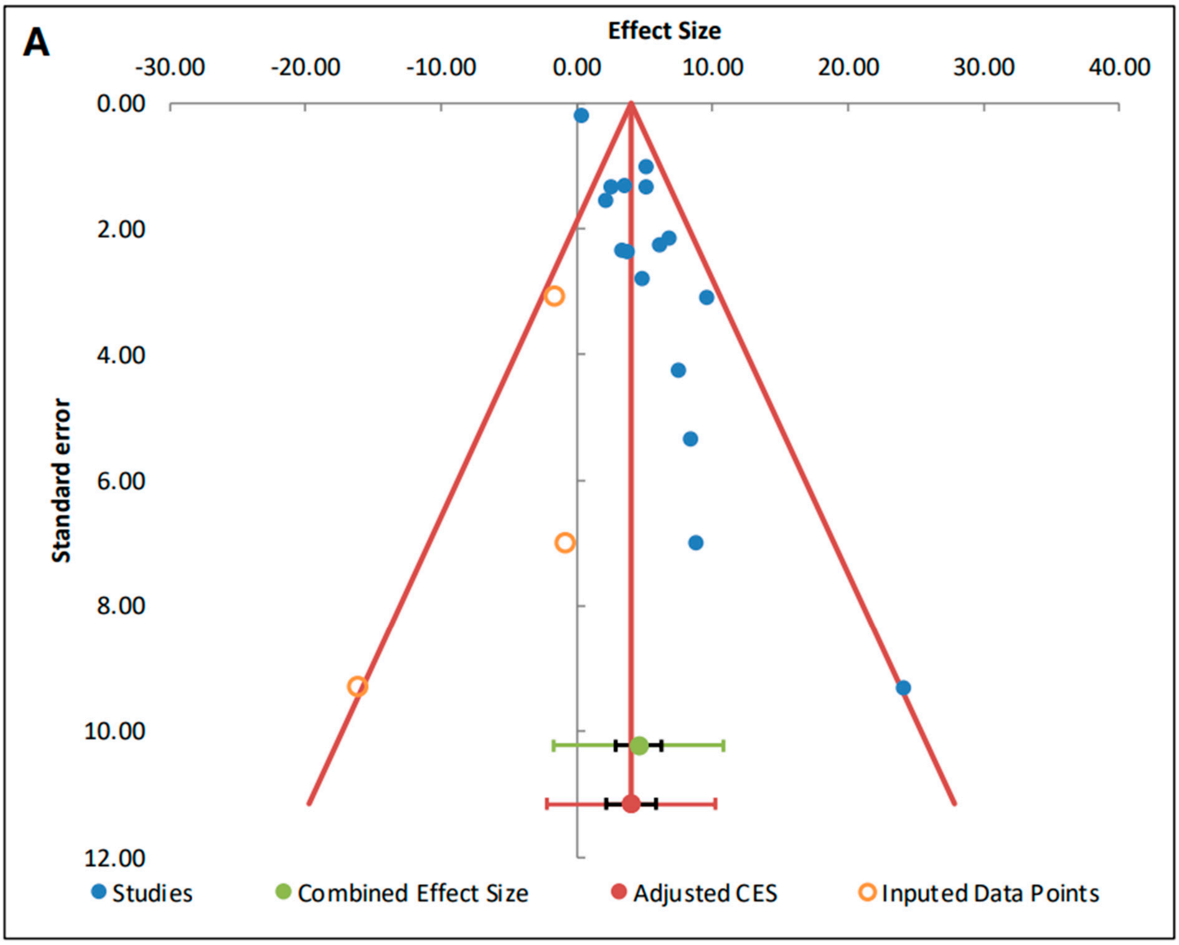

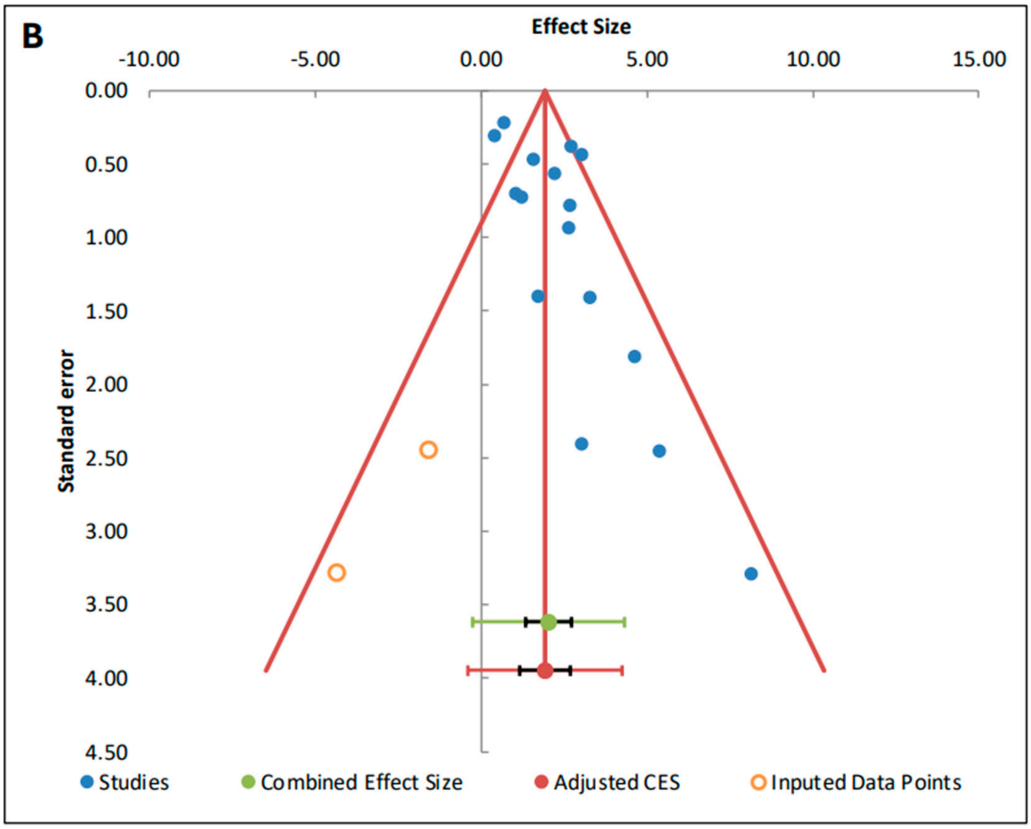

B
Effect Size
-10.00
-5.00
0.00
5.00
10.00
15.00
0.00
0.50
1.00
1.50
2.00
2.50
3.00
3.50
4.00
4.50
Standard error
Studies
Combined Effect Size
Adjusted CES
Inputed Data Points
